# Tests for the Assessment of Sport-Specific Performance in Olympic Combat Sports: A Systematic Review With Practical Recommendations

**DOI:** 10.3389/fphys.2018.00386

**Published:** 2018-04-10

**Authors:** Helmi Chaabene, Yassine Negra, Raja Bouguezzi, Laura Capranica, Emerson Franchini, Olaf Prieske, Hamdi Hbacha, Urs Granacher

**Affiliations:** ^1^Division of Training and Movement Sciences, Research Focus Cognition Sciences, University of Potsdam, Potsdam, Germany; ^2^Research Unit (UR17JS01) “Sport Performance & Health” Higher Institute of Sport and Physical Education of Ksar Said, Tunis, Tunisia; ^3^Department of Human Movement and Sport Sciences, University of Rome “Foro Italico”, Rome, Italy; ^4^Martial Arts and Combat Sports Research Group, School of Physical Education and Sport, Universidade de São Paulo, São Paulo, Brazil; ^5^Laboratoire Psychologie de la Perception, UMR Centre National de la Recherche Scientifique 8242, Paris, France; ^6^Cesam, EA 4260, Université de Caen Basse-Normandie, UNICAEN, Paris, France

**Keywords:** martial arts, validity, reliability, sensitivity, methodological quality, specific assessment

## Abstract

The regular monitoring of physical fitness and sport-specific performance is important in elite sports to increase the likelihood of success in competition. This study aimed to systematically review and to critically appraise the methodological quality, validation data, and feasibility of the sport-specific performance assessment in Olympic combat sports like amateur boxing, fencing, judo, karate, taekwondo, and wrestling. A systematic search was conducted in the electronic databases PubMed, Google-Scholar, and Science-Direct up to October 2017. Studies in combat sports were included that reported validation data (e.g., reliability, validity, sensitivity) of sport-specific tests. Overall, 39 studies were eligible for inclusion in this review. The majority of studies (74%) contained sample sizes <30 subjects. Nearly, 1/3 of the reviewed studies lacked a sufficient description (e.g., anthropometrics, age, expertise level) of the included participants. Seventy-two percent of studies did not sufficiently report inclusion/exclusion criteria of their participants. In 62% of the included studies, the description and/or inclusion of a familiarization session (s) was either incomplete or not existent. Sixty-percent of studies did not report any details about the stability of testing conditions. Approximately half of the studies examined reliability measures of the included sport-specific tests (intraclass correlation coefficient [ICC] = 0.43–1.00). Content validity was addressed in all included studies, criterion validity (only the concurrent aspect of it) in approximately half of the studies with correlation coefficients ranging from *r* = −0.41 to 0.90. Construct validity was reported in 31% of the included studies and predictive validity in only one. Test sensitivity was addressed in 13% of the included studies. The majority of studies (64%) ignored and/or provided incomplete information on test feasibility and methodological limitations of the sport-specific test. In 28% of the included studies, insufficient information or a complete lack of information was provided in the respective field of the test application. Several methodological gaps exist in studies that used sport-specific performance tests in Olympic combat sports. Additional research should adopt more rigorous validation procedures in the application and description of sport-specific performance tests in Olympic combat sports.

## Introduction

Amateur boxing, fencing, karate, judo, taekwondo, and wrestling represent popular combat sports. These combat sports are practiced in the whole world and constitute an important part of the Summer Olympic programme (International Olympic Committee, 2017a). Wrestling and fencing were already part of the first modern Olympic Games in 1896 for males. Females were included in 1924 for fencing and in 2004 for wrestling. In 1904, male amateur boxing was included in the official program of the Summer Olympic Games. It lasted until 2012, until female amateur boxing became part of the Olympic program. In 1964, judo was included in the Olympic program for males and in 1992 for females. Taekwondo was recognized as an Olympic sport in 2000 for both sexes and karate will be introduced for both sexes in the 2020 Olympic Games. In this regard and with reference to the growing interest in Olympic combat sports, it is important to advance scientific knowledge in performance testing to design specifically tailored training protocols and periodization models and to increase the likelihood of success in competition (Bridge et al., 2014; Chaabene et al., 2017a).

The main purposes of sport-specific testing can comprise talent identification and development of young athletes as well as the identification of strengths and weaknesses in young and elite athletes to be used for training purposes (Tabben et al., 2014; Chaabane and Negra, 2015). In addition, there is a consensus in the scientific literature on the importance of assessing physical and physiological qualities to optimize sport performance (Franchini et al., 2011a; Bridge et al., 2014; Chaabène et al., 2015c; Chaabene et al., 2017b) especially for those characterized by complex technical/tactical and physical/physiological demands like striking (e.g., karate, taekwondo, and amateur boxing; Chaabène et al., 2012b, 2015c; Bridge et al., 2014), grappling (e.g., judo and wrestling; Franchini et al., 2011a; Chaabene et al., 2017a), and weapon-based combat sports (e.g., fencing; Roi and Bianchedi, 2008).

However, prior to the design of a test protocol for sport-specific performance assessment, it is recommended to conduct a systematic needs analysis to identify the above-mentioned demands of the specific sport (Kraemer et al., 2012). More specifically, in the context of a need analysis, the metabolic, biomechanical, and injuries profile of the sport could be explored (Kraemer et al., 2012). With the systematically derived information on sport-specific demands from the needs analysis, adequate sport-specific performance tests can be designed and implemented into training practice. Information from these tests allows to identify strengths and weaknesses of athletes and to monitor how athletes' performance developed over time. These individualized performance profiles of athletes can be used for the planning of training protocols and periodization models. In Olympic combat sports, growing number of researchers have turned their attention to the development of valid sport-specific test protocols that are specifically tailored to the physical, physiological, technical and tactical demands of the respective sport discipline (Santos et al., 2010; Chaabène et al., 2012c; Tabben et al., 2014; Sant'Ana et al., 2017).

Even though there is a well-accepted advantage of sport-specific performance testing over the application of general physical fitness tests, there is no study available that systematically reviewed the methodological quality (e.g., sample size, inclusion/exclusion criteria, stability of testing conditions), validation data (i.e., reliability, validity, sensitivity), and feasibility (i.e., practicability) of the existing sport-specific tests related to Olympic combat sports. In fact, the majority of the available literature focused on the assessment of physical and physiological attributes of Olympic combat sport athletes in sports like amateur boxing (Chaabène et al., 2015c), fencing (Roi and Bianchedi, 2008), judo (Franchini et al., 2011a), karate (Chaabène et al., 2012b, 2015a), taekwondo (Bridge et al., 2014), and wrestling (Chaabene et al., 2017a). However, these tests assess general physical fitness qualities but not sport-specific performance. To the authors' knowledge, previous systematic reviews (Robertson et al., 2014; Hulteen et al., 2015) critically appraised the methodological quality and feasibility of performance tests in individual (e.g., golf, tennis, rock climbing) and team sports (e.g., football, rugby, volleyball) but not in Olympic combat sports. Therefore, the purpose of this study was to systematically review the available literature and to critically analyze the methodological quality, validation data, and feasibility of sport-specific tests in Olympic combat sports.

## Methods

The experimental approach comprehended five-steps (Khan et al., 2003): Step 1: Framing questions for the review; Step 2: Identification of relevant works; Step 3: Assessment of the quality of studies; Step 4: Summary the evidence; and Step 5: Interpretation of the findings.

### Step 1: framing questions for the review

The research question focused on sport-specific testing in Olympic combat sports (e.g., boxing, fencing, judo, karate, taekwondo, and wrestling). A Boolean search strategy was applied using the operators AND, OR. According to the main topic of the present study, the a-priori-specified inclusion criteria encompassed the following search syntax: [(“combat sport^*^” OR karate OR taekwondo OR “amateur boxing” OR judo OR wrestling OR fencing) AND (reliability OR validity OR sensitivity) AND (“physical fitness” OR “physiological characteristic^*^” OR “physical activity” OR “fitness test^*^” OR “motor assessment” OR “technical skill^*^” OR “gold standard”)].

### Step 2: identification of relevant works and data extraction

**Figure 1:**
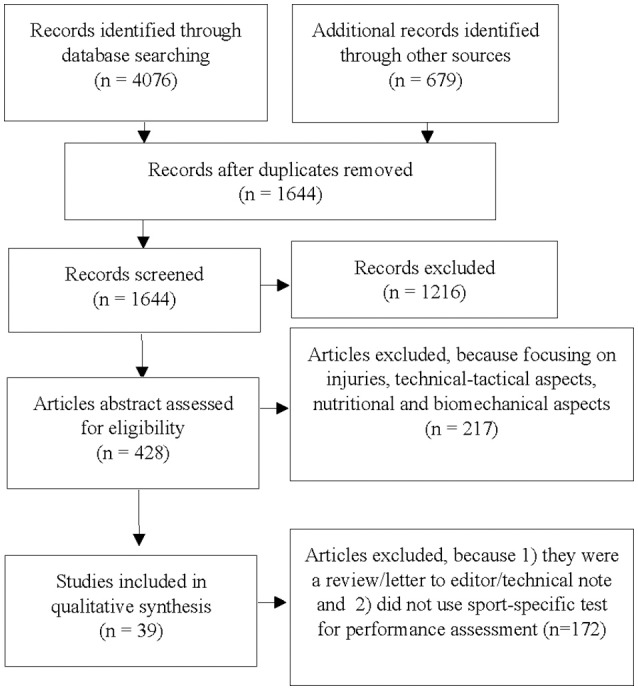
Flow of information through the different phases of the systematic review.

The present systematic review of the published literature was conducted based on the Preferred Reporting Items for Systematic Reviews and Meta-Analyses (PRISMA) guidelines (Moher et al., 2009). A comprehensive literature search of original manuscripts investigating was systematically performed on PubMed (MEDLINE), Google Scholar, and Science Direct. The search was limited to manuscripts published up to October 2017. In this study, the criteria for the inclusion of retrieved articles were: (i) written in English, (ii) published in peer-reviewed journals, (iii) focused on either on amateur boxing, fencing, judo, karate, taekwondo, wrestling, or a combination of these combat sports (iv) evaluate one aspect of the physical fitness and/or physiological characteristic through sport-specific testing, and (v) report at least one aspect of either reliability, validity, or sensitivity related to the applied test protocol. To allow the assessment of the methodological quality, only full-text sources were included, whereas abstracts and conference papers from annual meetings were not considered in the analysis. The first author (HC) coded the studies according to the selection criteria and eliminated duplicates. Relevant articles identified through the searching process were independently evaluated and assessed by two reviewers (e.g., HC and YN) who screened the titles, the abstracts, and the full texts to reach the final decision on the study inclusion or exclusion. In case of uncertainty or disagreement, a third expert was consulted. Additionally, the snowballing technique was applied to the reference lists of retrieved full-text articles to identify additional articles that were not included in the initial electronic search (Figure 1).

For each selected study included in the final list of scientific contributions considered eligible for the detailed examination, data were extracted and examined by two independent reviewers (HC and YN) who conducted data extraction following a predefined template. The template included sample size, demographic information including sex, age, and training background/expertise, type of combat sports, sport-specific test's name, objective, and duration, and validation data (e.g., reliability, validity, and sensitivity). After completion of data extraction, the two independent reviewers cross-checked the data to confirm their accuracy. Any conflicting results between reviewers resulted in a re-evaluation of the paper in question until a consensus was reached. It is worth noting that information related to the type of reliability (e.g., test-retest reliability and inter/intra-rater reliability; Currell and Jeukendrup, 2008; Robertson et al., 2014), validity (e.g., content or logical, criterion [concurrent/predictive], and construct [discriminative/convergent]) (Currell and Jeukendrup, 2008; Robertson et al., 2014), and sensitivity (Currell and Jeukendrup, 2008; Impellizzeri and Marcora, 2009) with the used corresponding statistical tools were specified.

Table 1 provides details of items related to the sport-specific tests' characteristics and feasibility. In addition, the most common statistical analysis approaches used to assess reliability were considered (Hopkins, 2000; Currell and Jeukendrup, 2008; Impellizzeri and Marcora, 2009), which encompassed: coefficient of variation (CV%), intra-class correlation coefficient (ICC), typical error of measurement (TEM), correlation coefficients (r), and 95% limits of agreement (LOA). Additionally, details related to the validity of the sport-specific testing were retrieved. Test sensitivity was assessed through comparing the smallest worthwhile change (SWC) and the typical error of measurement (TEM). Furthermore, the minimal detectable change (MDC_95%_), was also included (Hopkins, 2002).

**Table 1:** Details related to sport-specific tests' characteristics.

**Test characteristics**	**Definition**	**Assessment criteria**
**RELIABILITY**		
Test-retest reliability	The consistency of measurements, or of an individual's performance over repeated testing sessions or the absence of measurement error (Safrit and Wood, 1989) In the literature several terms have been used interchangeably with reliability such as repeatability, reproducibility, consistency, agreement, concordance, and stability (Atkinson and Nevill, 1998)	*Yes*: provided and shows good to excellent reliability *Partial*: provided, but (a) relative or absolute reliability not reported or (b) poor to average reliability level was established *Not provided*
Inter/intra-rater reliability	*Inter-rater*: the degree of agreement between assessments outcomes when undertaken by two or more testers (Baumgartner and Jackson, 1998) *Intra-rater*: the agreement among two or more trials undertaken by the same tester (Baumgartner and Jackson, 1998)	*Yes*: either or both examined *Partial*: reported, but (a) no reliability coefficient provided or (b) poor to average reliability shown *Not provided* *Not applicable*
**VALIDITY**		
Content	How well a specific test measures what it intends to measure (Currell and Jeukendrup, 2008; Thomas et al., 2015)	*Yes*: logical and/or content validity results established *Not provided*
Criterion (concurrent/predictive)	*Concurren*t: means that the performance protocol is correlated with a gold standard measure (Thomas et al., 2015) *Predictive*: Sport-specific outcome can predict sport performance (Thomas et al., 2015)	*Yes*: predictive or concurrent validity results established *Not provided*
Construct (discriminative/convergent)	*Construct*: whether a sport-specific test can measure a quality or attribute that cannot be operationalized *Discriminative*: ability to assess performers of different ability (as rated by another measure) (Russell et al., 2010; Streiner et al., 2015) *Convergent*: relation of a sport-specific test with another measure of the same construct or associated measures (Barrow et al., 1989; Russell et al., 2010)	*Yes*: discriminative and/or convergent validity results established *Not provided*
Responsiveness (sensitivity)	The sport-specific test is able to detect small, but meaningful changes in performance (Currell and Jeukendrup, 2008)	*Yes*: outcomes related to test responsiveness were established *Not provided*
Minimal detectable change	An estimation of the smallest change in score that can be detected objectively for a test, i.e., the amount by which the subject's performance needs to be changed to be sure the change is bigger than the measurement error (Haley and Fragala-Pinkham, 2006)	*Yes*: outcomes related to test minimal detectable change were established *Not provided*
**UTILITY AND LIMITATIONS**		
Feasibility and limitations	How well the sport-specific test is easy to be undertaken, administered, and scored (Stevens and Gibbins, 2002; Ali et al., 2008). Limitations relating to outcomes and interpretability of the test acknowledged and clarified in the study (Braeken et al., 2011)	*Yes*: practicability and limitations discussed *Partial*: practicability and limitations not fully discussed *Not provided*
Test background	Information relating to the expected use and context of the test detailed (Fliess-Douer et al., 2010)	*Yes*: information relating to test background established *Partial*: test background not fully discussed *Not provided*
Test duration	Expected or actual duration of the testing protocol established (Streiner, 1993)	*Yes*: expected or actual duration of test/trial established *Not provided*

### Step 3: assessment of the quality of studies

Based upon six criteria adapted from a risk-of-bias evaluation in a previously published review of tests examining sport-related skill outcomes (Robertson et al., 2014), two authors (HC and YN) carefully reviewed all eligible articles for quality appraisal (**Table 3**). These criteria included: (i) sample size, i.e., the number of participants included to establish validity/reliability/sensitivity of the sport-specific test; (ii) details related to study participants (e.g., sex, age, sport background/expertise, and anthropometric details); (iii) presence of clearly established inclusion/exclusion criteria; (iv) presence of familiarization session(s) prior to sport-specific testing or not; (v) presence of clearly established interval between test-retest assessments; and (vi) stability of testing conditions (i.e., whether testing equipment and environmental conditions remained stable between sessions or not) as well as participants between sessions.

### Step 4: summary the evidence

From each included study, the following information was extracted: author(s), year of publication, country, aim of the study, research design: Then, detailed tables reporting major characteristics of the selected studies were created.

### Step 5: interpretation of the findings

A synthesis of major findings reported in the included articles was submitted to a thematic analysis deemed relevant for generating inferences.

## Results

**Figure 2:**
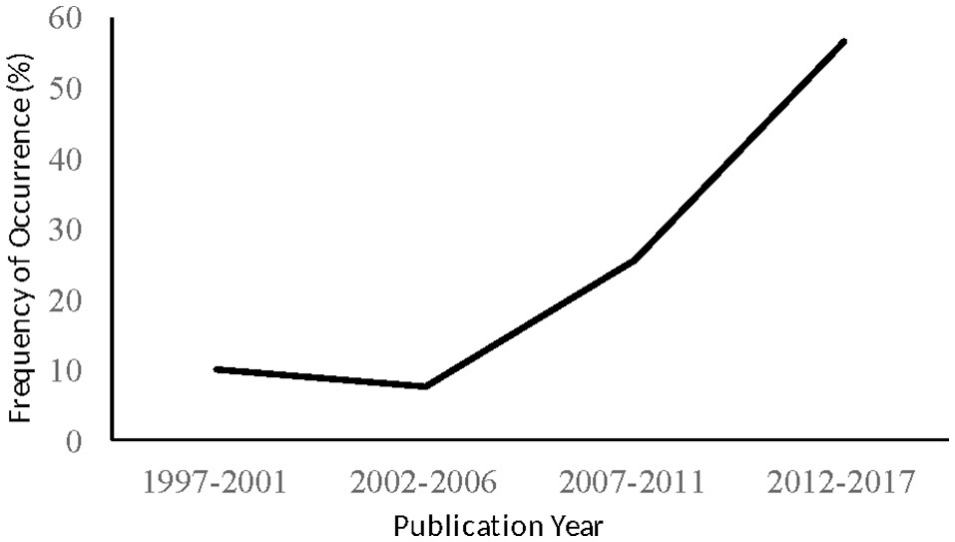
Frequency of Occurrence (%) of publication years of the scientific contributions included in the systematic literature review.

The preliminary systematic search resulted in 4,755 hits. After careful examination of titles and abstracts, 428 articles remained and were reviewed for eligibility. Full texts of these 428 articles were screened in regards of the previously defined inclusion/exclusion criteria. Finally, 39 articles were eligible to be included in this systematic review (Figure 1). Table 2 illustrates the main characteristics of the 39 eligible scientific contributions in terms of the applied sport-specific test, the tested physical and/or physiological attributes, the measured outcomes, and the number of female and male participants and their athletic background/expertise. Publication years ranged from 1997 (Utter et al., 1997) to 2017 (Araujo et al., 2017; Chaabene et al., 2017b; Sant'Ana et al., 2017), with an increasing trend starting from 2007 (Figure 2). Judo was represented in 15 articles (39%), whereas the relative picture for taekwondo, karate, fencing, wrestling, and amateur boxing was 8 (20%), 7 (18%), 4 (10%), 3 on (8%), and 2 (5%), respectively. The number of participants ranged from 5 to 219, with the majority of studies (74%) including <30 participants, whereas the proportion of studies including 30–49, 50–99, and >100 participants was 5, 13, and 3%, respectively. It is noteworthy that one study (Shiyan, 2011) did not include any information about participants' number. Detailed information on participants' characteristics were reported in 67% of the eligible studies with 31% providing a partial description, and another 2% lacking information regarding the recruited participants (Table 2). The proportion of studies including athletes competing at national or international level was 41%, that including mixed samples of national/international and regional/sub-elite level athletes was 33%, and that recruiting club/regional level participants was 26%. The sex representation of the participants was 61% for male athletes, 5% for female athletes, and 28% for both sexes. No information on the sex of the participants was present for 6% of the studies.

**Table 2:** Characteristics of the eligible studies.

**Combat sport discipline**	**References**	**Test name**	**Physical/physiological attribute(s) tested**	**Outcomes (measure unit)**	**Participants' athletic background**
Karate	Chaabène et al., 2015b	Karate specific aerobic test	Maximal aerobic power	Time (s)	Male international level (*n* = 12) Female international level (*n* = 3)
Karate	Chaabène et al., 2012c	Karate specific aerobic test	Maximal aerobic power	Time (s)	Male national level (*n* = 16)
Karate	Chaabène et al., 2012b	Karate specific aerobic test	Maximal aerobic power	Time (s)	Male national and regional level (*n* = 43)
Karate	Sertić et al., 2011	Movement change in karate position (MKUKS)	Agility	Time (s)	Male club level (*n* = 65)
Karate	Tabben et al., 2014	Karate Specific Test	Maximal aerobic power	VO_2peak_ (ml/ kg/ min) Time (s)	Male international Level (*n* = 14) Female international Level (*n* = 3)
Karate	Nunan, 2006	Karate specific aerobic test	Maximal aerobic power	Time (s) VO_2peak_ (ml/ kg/ min)	Male national Level (*n* = 5)
Karate	Sterkowicz and Franchini, 2009	Hip turning speed test Speed punches test Flexibility test Rapid kicks test Agility test Evasion action test	Speed and coordination Speed and coordination Flexibility Speed and coordination Agility Agility	Time (s) Time (s) Time (s) Flexibility index Time (s) Time (s)	Male Kyokushin of various competitive levels (*n* = 219)
Taekwondo	Araujo et al., 2017	Specific Taekwondo exercise test (TKDtest)	Maximal aerobic power and capacity	VO_2peak_ (ml/ kg/ min) HR_peak_ (batt/min) Ventilatory thresholds (VT1 and VT2 [ml/ kg/ min])	Male black belt athletes (*n* = 14)
Taekwondo	Sant'Ana et al., 2017	Progressive specific taekwondo test (PSTT)	Maximal aerobic power	VO_2peak_ (ml/ kg/ min)	Male national and regional level (*n* = 18)
Taekwondo	Chen et al., 2015	Dual task test	Reaction time and skill proficiency in roundhouse kick	Time (s)	Male elite athletes (*n* = 12) Male sub-elite athletes (*n* = 12)
Taekwondo	Rocha et al., 2016	Taekwondo specific anaerobic test (TSAT)	Anaerobic power Anaerobic capacity Fatigue index	W and W/kg W and W/kg %	Male elite level (n = 17)
Taekwondo	Sant'Ana et al., 2014	Taekwondo anaerobic test (TAT)	Anaerobic power and capacity: AKC MKT BKT MKI HKI	Number of Repetition (n) Time (s) Time (s) Kick impact (g) Kick impact (g)	Male elite level (n = 10)
Taekwondo	Oliveira et al., 2015	Adapted anaerobic kick test (AAKT)	Anaerobic power and capacity: Higher kick frequency (higher frequency of kicks performed during 3 s) Lower kick frequency (lower frequency of kicks performed during 3 s)	Kicks/s Kicks/s Kicks/s % Time (s)	Male club level (*n* = 10) Female club level (*n* = 5)
			Average kick frequency (average frequency of kicks performed during 30 s) Fatigue index (percentage reduction of the maximum frequency kick to minimum frequency kick) Time to higher kick frequency (time from start to the higher frequency of kicks performed during 3 s)		
Taekwondo	da Silva Santos and Franchini, 2016	Frequency speed of kick test (FSKT) Multiple frequency speed of kick test (MFSKT)	Anaerobic power and capacity	Number of repetition (n)	Male black-belt (*n* = 4) Female black-belt (*n* = 4)
Taekwondo	Chaabene et al., 2017b	Taekwondo-specific agility test	Planned agility	Time (s)	Male elite level (*n* = 20) Female elite level (*n* = 7)
Amateur boxing	Smith et al., 2000	Sport-specific boxing dynamometer	Maximal punching force	Newton (N)	Male elite level (*n* = 7) Male Intermediate level (*n* = 8) Male novice level (*n* = 8)
Amateur boxing	Obminski et al., 2011	Shut put test	Explosive strength	Centimeter (cm)	Male elite level (*n* = 6) Female elite level (*n* = 7)
Judo	Santos et al., 2010	Santos test	Maximal aerobic power and capacity	VO_2peak_ (ml/ kg/ min) HR (batt/min) Blood lactate (mmol/l)	Male national level (*n* = 8)
Judo	Santos et al., 2012	Santos test	Maximal aerobic power and capacity	VO_2peak_ (ml/ kg/ min) HR (batt/min) Blood lactate (mmol/l)	Female elite level (*n* = 8)
Judo	Santos et al., 2011	Santos test	Maximal aerobic power and capacity	VO_2peak_ (ml/ kg/ min) HR (batt/min) Blood lactate (mmol/l)	Male elite level (*n* = 8)
Judo	Sogabe et al., 2015	Uchikomi shuttle run test	Judo-specific endurance	Number of Uchikomi (n) HR (batt/min) Rating of perceived exertion	Competitive athletes Gender NP (*n* = 18)
Judo	Tavra et al., 2016	Santos test Uchi-komi fitness test Ten station judo ability test Special judo fitness test (SJFT)	Maximal aerobic power and capacity Anaerobic capacity Judo specific ability Anaerobic performance	VO_2peak_ (ml/ kg/ min) HR (batt/min) Blood lactate (mmol/l) Nombre d'Uchi-Komi Time (s) SJFT index	Female elite and sub-elite level (*n* = 14)
Judo	Sterkowicz and Franchini, 2001	Special judo fitness test (SJFT)	Anaerobic performance	SJFT index	Male elite level (*n* = 33) Male novice level (*n* = 47)
Judo	Franchini et al., 1998	Special judo fitness test (SJFT)	Anaerobic performance	SJFT index	Male regional and state level (*n* = 17)
Judo	Franchini et al., 2011b	*Judogi* grip strength test	Strength endurance	Time (s) Number of repetition (n)	Male elite level (*n* = 16) Male regional level (*n* = 12)
Judo	Lidor et al., 2005	Judo-specific test	Judo specific ability	Time (s)	Male club level (*n* = 10)
Judo	Azevedo et al., 2007	Lactate minimum intensities for judo	Aerobic capacity	HR (batt/min) Blood lactate (mmol/l) Drills/s	Male regional and international level (*n* = 6)
Judo	Morales et al., 2016	Randori maximal time to exhaustion	Aerobic capacity	Time (min)	Male international level (*n* = 7) Female international level (*n* = 4)
Judo	Del Vecchio et al., 2014	Hikidashi uchi-komi test	Anaerobic performance	Repetition (n)	*First study:* Male regional and state level (*n* = 10) *Second study:* Male regional and state level (*n* = 24) and female regional and state level (*n* = 6)
Judo	de Azevedo et al., 2014	Judo specific incremental test (JSIT)	Aerobic capacity	Respiratory compensation threshold VO_2_ (ml/ kg/ min), HR (batt/min) blood lactate (mmol/l)	Male well-trained (*n* = 8)
Judo	Almansba et al., 2012	Uchikomi Fitness Test (UFT)	Judo specific fitness	Number of Uchi-komi (n) HR (batt/min)	Male of various competitive level (*n* = 7)
Judo	Franchini et al., 2005	Special judo fitness test (SJFT)	Anaerobic performance	SJFT index	Male elite level (*n* = 23) Male non-elite level (*n* = 53)
Wrestling	Shiyan, 2011	Special endurance test	Special endurance	Special endurance coefficient	Elite level Gender NP (*n* = NP)
Wrestling	Wright et al., 2015	Sandbag test	Sport-specific fitness	Score (T/T [time to finish each round/number of throws]) Fatigue (%)	Male national level (*n* = 15)
Wrestling	Utter et al., 1997	Pittsburgh wrestling performance test	Sport-specific performance	Time (s)	Male university level (*n* = 7)
Fencing	Bottoms et al., 2013	Laboratory based protocol (LBP)	Sport-specific performance	HR (batt/min) Rating of perceived exertion	Male club level (*n* = 6)
Fencing	Weichenberger et al., 2012	Fencing-specific endurance test (FET)	Aerobic capacity	HR (batt/min) Blood lactate (mmol/l)	*Validation study*: elite level male (n = 15) and elite level female (*n* = 13) *Discrimination study*: male international level fencers (*n* = 19) and male national level fencers (*n* = 20)
Fencing	Turner et al., 2016	Elite level male (n = 15) and elite level female (n = 13)	Change of direction speed	Time (s)	Male elite level (*n* = 49) Female elite level (21)
Fencing	Tsolakis and Vagenas, 2010	Time of lunge test Time of shuttle test	Muscular power change of direction speed	Time (s) Time (s)	Male and female elite level (*n* = 14) Male and female sub-elite level (*n* = 19)

*NP, not provided; AKC, number of kicking cycles; MKT, mean kicking time; BKT, best kicking time; MKI, mean kicking impact; HKI, highest kicking impact; HR, heart rate; VO_2peak_, peak oxygen uptake; W, watt; kg, kilogram, s, second*.

### Methodological quality of the eligible studies

The methodological characteristics of the included studies are shown in Table 3. Whilst only 28% of the included studies provided detailed inclusion/exclusion criteria, 26% provided a partial description and 46% did not include any information. Only 38% of the studies scheduled one or more familiarization sessions for their participants and 2% provided partial details on this relevant aspect, whereas the majority of the studies (60%) presented a lack of information. Studies focusing on reliability (61%) included intervals between experimental trials ranging from 1 day to 11 weeks, with 57% of them adopting a 1-week interval. However, information on this issue was lacking in 39% of studies. Only 28% of the studies provided detailed information on stability of testing conditions, with 59% of them not presenting any information and 13% providing partial information.

**Table 3:** Methodological quality of eligible studies.

**Combat sport discipline**	**References**	**Sample size**	**Details related to study participants**	**Inclusion/exclusion criteria**	**Acquainting sessions**	**Test-retest interval**	**Stability of testing conditions**
Karate	Chaabène et al., 2015b	§	Yes	Yes	Yes	Reliability NS	Yes
Karate	Chaabène et al., 2012c	§	Yes	Yes	Yes	1 week	Yes
Karate	Chaabène et al., 2012b	§§	Yes	Yes	Yes	1 week	Yes
Karate	Sertić et al., 2011	§§§	Partial	NP	Partial	NP	NP
Karate	Tabben et al., 2014	§	Yes	Yes	Yes	1 week	Yes
Karate	Nunan, 2006	§	Yes	Partial	NP	1 week	NP
Karate	Sterkowicz and Franchini, 2009	§§§§	Partial	NP	NP	NP	NP
Taekwondo	Araujo et al., 2017	§	Yes	Yes	Yes	2–7 days	Yes
Taekwondo	Sant'Ana et al., 2017	§	Yes	Partial	NP	Reliability NS	NP
Taekwondo	Chen et al., 2015	§	Yes	Partial	NP	1 days	NP
Taekwondo	Rocha et al., 2016	§	Partial	Partial	Yes	1 week	Yes
Taekwondo	Sant'Ana et al., 2014	§	Yes	Yes	NP	Reliability NS	NP
Taekwondo	Oliveira et al., 2015	§	Partial	NP	NP	Reliability NS	NP
Taekwondo	da Silva Santos and Franchini, 2016	§	Partial	NP	NP	11 weeks	NP
Taekwondo	Chaabene et al., 2017b	§	Yes	Partial	Yes	1 week	Yes
Amateur boxing	Smith et al., 2000	§	Yes	Yes	Yes	NP	NP
Amateur boxing	Obminski et al., 2011	§	Partial	NP	Yes	NP	NP
Judo	Santos et al., 2010	§	Yes	Yes	NP	1 week	Yes
Judo	Santos et al., 2012	§	Yes	Yes	NP	1 week	Yes
Judo	Santos et al., 2011	§	Yes	Yes	Yes	NP	Partial
Judo	Sogabe et al., 2015	§	Partial	Partial	NP	Reliability NS	NP
Judo	Tavra et al., 2016	§	Yes	Yes	NP	Reliability NS	Yes
Judo	Sterkowicz and Franchini, 2001	§§§	Yes	NP	NP	Reliability NS	NP
Judo	Franchini et al., 1998	§	Partial	NP	NP	Reliability NS	NP
Judo	Franchini et al., 2011b	§	Yes	NP	NP	Reliability NS	NP
Judo	Lidor et al., 2005	§	Yes	Partial	NP	Reliability NS	NP
Judo	Azevedo et al., 2007	§	Yes	NP	NP	Reliability NS	NP
Judo	Morales et al., 2016	§	Yes	NP	NP	48–72 h	NP
Judo	Del Vecchio et al., 2014	§§	Yes	NP	NP	48 h	Partial
Judo	de Azevedo et al., 2014	§	Yes	NP	Yes	Reliability NS	Partial
Judo	Almansba et al., 2012	§	Yes	NP	NP	48 h	Yes
Judo	Franchini et al., 2005	§§§	Partial	NP	NP	Reliability NS	NP
Wrestling	Shiyan, 2011	NP	NP	NP	NP	Reliability NS	NP
Wrestling	Wright et al., 2015	§	Yes	Partial	Yes	NP	Partial
Wrestling	Utter et al., 1997	§	Partial	NP	Yes	NP	NP
Fencing	Bottoms et al., 2013	§	Partial	NP	NP	Reliability NS	NP
Fencing	Weichenberger et al., 2012	§§	Partial	NP	NP	Reliability NS	NP
Fencing	Turner et al., 2016	§§§	Yes	Partial	Yes	NP	Partial
Fencing	Tsolakis and Vagenas, 2010	§§	Yes	Partial	Yes	NP	NP

*NP, Not provided; NS, not studied; §, less than 30 participants; §§, between 30 and 49 participants; §§§, between 50 and 99 participants; §§§§, more than 100*.

### Reliability

Table 4 illustrates detailed information on validation data of the respective sport-specific tests. From the 39 included studies, 51% reported reliability data of the sport-specific test. Test-retest reliability was examined in 85% of the studies that conducted reliability analysis, with 15% dealing with inter/intra-rater reliability. The ICC (range 0.43–1) was used in 80% of the studies and constituted, therefore, the most frequently applied statistical approach to assess reliability in sport-specific tests of Olympic combat sports. The SEM was used in 30% of the identified studies, the CV in 5%, the 95% LoA in 15%, and correlation coefficients in 5% of the studies. Other statistical approaches (i.e., paired sample *t*-tests and Wilcoxon signed rank tests) were used to establish reliability in 15% of the studies. Only 40% of the studies applied mixed statistical approaches to examine relative and absolute reliability as recommended by previous research (Atkinson and Nevill, 1998) and the greater part of them (60%) adopted only one statistical approach (most often ICC [67%]).

**Table 4:** Sport specific tests characteristics.

**Combat sport discipline**	**References**	**Reliability type (statistical approach used and results)**	**Validity type (statistical approach used)**	**Sensitivity (statistical approach used)**	**Minimal detectable change**	**Feasibility and limitations**	**Test background**	**Test duration (s)**
Karate	Chaabène et al., 2015b	Test –retest NP Inter/intra-rater reliability NP	Yes Content Criterion (concurrent) (r = 0.14)	NP	NP	Yes	Yes	Yes
Karate	Chaabène et al., 2012c	Yes (test-retest) (ICC = 0.982) (SEM = 29.37 s) Inter/intra-rater reliability NA	Yes Content	Yes SWC	Yes	Yes	Yes	Yes
Karate	Chaabène et al., 2012b	Yes (test-retest) (ICC = 0.98) (SEM = 28.5 s) (95% LOA = 9.5 ±78.8 s) Inter/intra-rater reliability NA	Yes Content Construct (discriminative) ROC analysis: area under the ROC curve = 0.86	NP	NP	Yes	Yes	Yes
Karate	Sertić et al., 2011	Yes Test –retest NP (Inter/intra-rater reliability ICC = 0.96)	Yes Content Criterion (concurrent) (*r* = 0.36–0.60)	NP	NP	NP	NP	Yes
Karate	Tabben et al., 2014	Yes (test-retest) (ICC = 0.99) (SEM = 2.22 s) Inter/intra-rater reliability NA	Yes Content Criterion (concurrent) (r = 0.81–0.83)	Yes SWC	Yes	Yes	Yes	Yes
Karate	Nunan, 2006	Yes (test-retest) (Wilcoxin signed-rank test) Inter/intra-rater reliability NA	Yes Content	NP	NP	Yes	Yes	Yes
Karate	Sterkowicz and Franchini, 2009	Yes (test-retest) (ICC = 0.82–0.96) Inter/intra-rater reliability NA	Yes Content Criterion (concurrent) (*r* = 0.31–0.43)	Yes (NP)	NP	Partial	Yes	Yes
Taekwondo	Araujo et al., 2017	Yes (test- retest) (ICC = 0.70–0.85) Inter/intra-rater reliability NA	Yes Content Criterion (concurrent) (Paired *T*-test; 2-way ANOVA; 95% LOA)	NP	NP	Yes	Yes	Yes
Taekwondo	Sant'Ana et al., 2017	Test-retest NP Inter/intra-rater reliability NP	Yes Content Criterion (concurrent) (Paired *T*-test and 95% LOA)	NP	NP	Yes	Yes	Yes
Taekwondo	Chen et al., 2015	Yes (test-retest) (ICC = 0.43–0.95) Inter/intra-rater reliability NP	Yes Content Construct (discriminative) (independent sample *t*-test).	NP	NP	Partial	Yes	Yes
Taekwondo	Rocha et al., 2016	Yes (test-retest) (ICC = 0.80–0.93) Inter/intra-rater reliability NA	Yes Content Criterion (concurrent) (*r* = 0.55–0.88) (95% LOA)	NP	NP	Partial	Yes	Yes
Taekwondo	Sant'Ana et al., 2014	Test-retest NP Inter/intra-rater reliability NP	Yes Content Criterion (concurrent) (*r* = 0.70–0.89)	NP	NP	Partial	Yes	Yes
Taekwondo	Oliveira et al., 2015	Test-retest NP Inter/intra-rater reliability NP	Yes Content Criterion (concurrent) (*r* = 0.31–0.86)	NP	NP	Partial	Partial	Yes
Taekwondo	da Silva Santos and Franchini, 2016	Yes Test-retest NP (intra-rater) (ICC = 0.99–1.0)	Yes Content	Yes (SWC)	NP	Yes	Yes	Yes
Taekwondo	Chaabene et al., 2017b	Yes Test-retest (ICC = 0.97) (SEM = 1.82%) Inter/intra-rater reliability NP	Yes Content Criterion (concurrent) (*r* = 0.71) Construct (discriminative) (ROC analysis: area under the ROC curve = 0.0.94)	Yes (SWC)	Yes	Yes	Yes	Yes
Amateur boxing	Smith et al., 2000	Test-retest NP Inter/intra-rater reliability NP	Yes Content Construct (discriminative) (Two-way repeated ANOVA)	NP	NP	NP	NP	NP
Amateur boxing	Obminski et al., 2011	Test-retest NP Inter/intra-rater reliability NP	Yes Content	NP	NP	Partial	Partial	NP
Judo	Santos et al., 2010	Yes (test-retest) (Paired sample *T*-test) Inter/intra-rater reliability NA	Yes Content Criterion (concurrent) (Paired sample *T*-test)	NP	NP	Partial	Yes	NP
Judo	Santos et al., 2012	Yes (test-retest) (Paired sample *T*-test) Inter/intra-rater reliability NA	Yes Content Criterion (concurrent) (Paired sample *T*-test)	NP	NP	Partial	Yes	NP
Judo	Santos et al., 2011	Test-retest NP Inter/intra-rater reliability NP	Yes Content Criterion (concurrent) (Paired sample *T*-test)	NP	NP	Partial	partial	NP
Judo	Sogabe et al., 2015	Test-retest NP Inter/intra-rater reliability NP	Yes Content Criterion (concurrent) (*r* = −0.56)	NP	NP	Yes	Partial	NP
Judo	Tavra et al., 2016	Test-retest NP Inter/intra-rater reliability NP	Yes Content Construct (discriminative) (Independent *t*-test)	NP	NP	Partial	Partial	Yes
Judo	Sterkowicz and Franchini, 2001	Test-retest NP Inter/intra-rater reliability NP	Yes Content Construct (discriminative) (2-way ANOVA)	NP	NP	Partial	Yes	Yes
Judo	Franchini et al., 1998	Test-retest NP Inter/intra-rater reliability NP	Yes Content Construct (discriminative) (one way ANOVA)	NP	NP	NP	Partial	Yes
Judo	Franchini et al., 2011b	Test-retest NP Inter/intra-rater reliability NP	Yes Content Construct (discriminative) (ANCOVA)	NP	NP	Partial	Yes	Yes
Judo	Lidor et al., 2005	Test-retest NP Inter/intra-rater reliability NP	Yes Content Criterion (predictive) (*r* = −0.11–0.31)	NP	NP	Partial	Yes	NP
Judo	Azevedo et al., 2007	Test-retest NP Inter/intra-rater reliability NP	Yes Content Criterion (concurrent) (Wilcoxon signed rank test)	NP	NP	Partial	Yes	Yes
Judo	Morales et al., 2016	Yes Test-retest (ICC = 0.91; SEM = 0.53 min, and 95% LOA) Inter/intra-rater reliability NA	Yes Content Criterion (concurrent) (*r* = 0.66)	NP	NP	Partial	Yes	Yes
Judo	Del Vecchio et al., 2014	Yes Test-retest (ICC = 0.71–0.93; 95% LOA) Inter/intra-rater reliability NA	Yes Content Criterion (concurrent) (*r* = 037–0.80) Construct (discriminative) (independent sample *t*-test)	NP	NP	Partial	Yes	Yes
Judo	de Azevedo et al., 2014	Test-retest NP Inter/intra-rater reliability NP	Yes Content Criterion (concurrent) (paired sample *t*-test; *r* = 0.90)	NP	NP	Partial	Yes	NP
Judo	Almansba et al., 2012	Yes Test-retest (ICC = 0.88–0.99; SEM = 0.6–2.1%) Inter/intra-rater reliability NA	Yes content	NP	NP	Partial	Yes	Yes
Judo	Franchini et al., 2005	Test-retest NP Inter/intra-rater reliability NP	Yes Content Construct (discriminative) (ANCOVA)	NP	NP	NP	NP	Yes
Wrestling	Shiyan, 2011	Test-retest NP Inter/intra-rater reliability NP	Yes Content	NP	NP	Partial	Partial	NP
Wrestling	Wright et al., 2015	Yes (test-retest) (ICC = 0.95–0.96) (CV = 9.3–34.3%) Inter/intra-rater reliability NA	Yes Content	NP	NP	Yes	Yes	Yes
Wrestling	Utter et al., 1997	Yes (test-retest) (*r* = 0.97) Inter/intra-rater reliability NA	Yes Content	NP	NP	Yes	Yes	Yes
Fencing	Bottoms et al., 2013	Test-retest NP Inter/intra-rater reliability NP	Yes Content (two-way ANOVA)	NP	NP	Partial	Yes	Yes
Fencing	Weichenberger et al., 2012	Test-retest NP Inter/intra-rater reliability NP	Yes Content Criterion (concurrent) (r = 0.30-0.31) Construct (discriminative) (independent sample *t*-test)	NP	NP	Yes	Yes	NP
Fencing	Turner et al., 2016	Yes Test-retest NP Intra-rater (ICC = 0.95)	Yes Content Criterion (concurrent) (*r* = −0.65-−0.41) (Regression analysis)	NP	NP	Yes	Yes	Yes
Fencing	Tsolakis and Vagenas, 2010	Yes Test-retest (ICC = 0.93–0.98) Inter/intra-rater reliability NA	Yes Content Construct (discriminative) (independent sample *t*-test)	NP	NP	NP	NP	Yes

*NP, not provided; NA, not applicable; ICC, intraclass correlation coefficient; SEM, standard error of measurement; CV, coefficient of variation; SWC, smallest worthwhile change; r, correlation coefficient; LOA, limits of agreement: ROC, Receiving operator characteristic. ANOVA, Analysis of variance; ANCOVA, Analysis of covariance*.

### Validity and sensitivity

All reviewed studies presented at least one aspect of test validity. Of note, content validity was addressed in all identified studies. Criterion validity was determined in 54% of the eligible studies, with 95% addressing concurrent validity (*r* = −0.41 to 0.90) and 5% predictive validity. From the studies that addressed concurrent validity, 60% applied correlation coefficients only, whereas 10% used mixed correlation coefficients with other methods (e.g., 95% LOA, regression analysis), and 30% applied other approaches (e.g., Wilcoxon signed rank test, paired sample *T*-test, 95% LOA). Construct validity was examined in 31% of the identified 39 studies, with the discriminative side of it (i.e., the ability of the sport-specific test to differentiate performance according to expertise level) being the most important aspect in all studies. This was realized by computing receiver operator characteristic (ROC) analyses, independent sample *t*-tests, and two-way repeated measures ANOVA. Only 23% of the identified studies addressed content validity, 77% examined mixed aspects (e.g., content with criterion validity, content with construct validity or content with the criterion and construct validity). One of the three sport-specific testing aspects (e.g., content, criterion, and construct validity) was investigated in only 8% of the studies. The sensitivity of sport-specific testing was investigated in 13% of the reviewed studies. These studies mainly calculated the SWC and compared it with SEM (Chaabène et al., 2012a; Chaabene et al., 2017b; Tabben et al., 2014) and one study (da Silva Santos and Franchini, 2016) used data recorded after a 9-week training period to appraise sensitivity of the respective sport-specific test. The MDC_95%_ of the sport-specific test was addressed in 8% of the reviewed studies.

### Utility and limitations

Feasibility and methodological limitations of the sport-specific tests were sufficiently explored in 36% of the included studies, with 51% providing partial details and 13% ignoring this aspect. Information related to the expected use and context of the test was adequately pointed out in 72% of the included studies. Eighteen percent reported limited information on this aspect and 10% ignored this relevant sport-specific aspect. Sport-specific test duration was described in detail in 74% of the included studies and the remaining 26% did not report any information on this issue.

## Discussion

The main goal of this study was to examine the methodological quality, validation data, and feasibility of sport-specific tests in Olympic combat sports. This is the first study detailing the different methodological approaches adopted so far with sport-specific tests in Olympic combat sports. Results of this study highlighted: (1) emerging academics conversation on sport-specific tests in Olympic combat sports; (2) a disparity in the gender representation of participants; and (3) several methodological gaps in the study of sport-specific testing in Olympic combat sports.

Since 2006 a substantial increase in publication activity has been observed, coherently with the quest of sport-specific testing procedures to evaluate Olympic combat sport athletes. At present, research in sport-specific testing of Olympic combat sports could be considered entering its intermediate stage (Edmondson and McManus, 2007), being characterized by not fully established theories and several methodological shortcomings. In particular, a lack of a “gold standard” technique to assess sport-specific outcomes in peculiar combat sport contexts and valid and reliable tools suited to large-scale assessments limits the generalizability of findings. Furthermore, in considering that ~40% of the eligible studies focused on judo, the need to develop valid sport-specific tests for athletes practicing other Olympic combat sports emerged.

Overall, a major challenge for the interpretation of sport-specific test data to be used for training periodization is due to methodological limits. Even though researchers have attempted to develop and validate sport-specific tests in Olympic combat sports, future studies should carefully address methodological aspects. More specifically, further research should focus on i) tests that accurately reflect athletes' sport-specific performance strengths and weaknesses and ii) present good level of predictive validity. Particularly, special attention should be directed toward the recruitment of a wide range of athletes, detailing clear inclusion/exclusion criteria of participants, and presenting sufficient description of their characteristics such as anthropometrics, age, and expertise level. This issue is crucial to guarantee the test-specificity for different populations of athletes, which allows coaches to programme sound individualized training plans. Furthermore, clear and comprehensive information on test procedures is needed so the protocol can be easily reproduced (Morrow et al., 2015). Another aspect to be considered is the provision of information on test-retest reliability, including test-retest intervals, intra- and inter-rater reliability, and the stability of testing conditions, which could determine problems in the interpretation of results (Atkinson and Nevill, 1998; Morrow et al., 2015).

In general, validity, reliability and sensitivity are basic criteria for a test able to assess sport performances. When examining validation data, approximately half of the included studies examined reliability of the sport-specific test using test-retest as the most frequently applied reliability aspect and ICCs were most often computed (80%). Content validity was addressed in all identified studies. Criterion validity and more specifically the concurrent side was assessed in approximately half of the studies, while construct validity received less attention (31% of the studies). Of note, predictive validity was surprisingly neglected. In fact, only one study that examined this test characteristic (Lidor et al., 2005) has been identified. Additionally, few studies examined test sensitivity (13%). Feasibility and methodological limitations were partially reported and/or ignored in the majority of the reviewed (64%) studies. Detailed information related to the expected use and context of the protocol were either partially reported or ignored in 28% of the studies.

### Methodological quality of the included studies

One major point related to the methodological quality is the limited sample size recruited in the majority of the reviewed studies. It is consensual that sample size is the most critical aspect decoding study's outcome quality and applicability (Hopkins et al., 2009). In this contest, 5 studies included between 50 and 99 participants (Sterkowicz and Franchini, 2001; Sertić et al., 2011; Weichenberger et al., 2012; Turner et al., 2016), one study included more than 100 participants (Sterkowicz and Franchini, 2009), and one study did not provide participants' number (Shiyan, 2011). This observation seems to be due to the limited number of coaches agreeing their athletes to be involved in such studies. One more issue that may prevent and/or question sport-specific tests to be applied with other population, for instance, amateur and beginner practitioners, is the recruitment of national/international level athletes in most of the studies. Compared with males, females were recruited in two studies (Santos et al., 2012; Tavra et al., 2016), with 28% of the studies recruiting combat sport athletes of both sexes (Tsolakis and Vagenas, 2010; Obminski et al., 2011; Weichenberger et al., 2012; Del Vecchio et al., 2014; Tabben et al., 2014; Chaabène et al., 2015b; Chaabene et al., 2017b; Oliveira et al., 2015; da Silva Santos and Franchini, 2016; Morales et al., 2016; Turner et al., 2016). This seems to be mainly due to the limited interest and/or opportunity of females in combat sports, as only recently female competitions were included in Olympic boxing and wrestling, for instance. Also, cultural constraints to female participation in combat sports (Miarka et al., 2011) may determine the gender-related discrepancies in the sport sciences literature, which does not mirror the increased participation of women in the last editions of the Olympic Games (International Olympic Committee, 2017b). Therefore, the sports scholars are urged to intensify their efforts to bridge this imbalance between women's sport participation and scientific information on this specific population.

Details related to the recruited participants were either partially reported or ignored in 33% of the studies (Utter et al., 1997; Franchini et al., 1998, 2005; Sterkowicz and Franchini, 2009; Obminski et al., 2011; Sertić et al., 2011; Shiyan, 2011; Weichenberger et al., 2012; Bottoms et al., 2013; Oliveira et al., 2015; Sogabe et al., 2015; da Silva Santos and Franchini, 2016; Rocha et al., 2016). This issue markedly affects the quality of the study and prevents the sport-specific test of being replicated and used. There is a lack (26% of the studies) and most often absence (46% of the studies) of any inclusion/exclusion criteria and only 28% of the studies sufficiently detailed this aspect. Therefore, future investigations are encouraged to consider clarifying this important research aspect. Despite their relevance in reducing measurement error, mainly systematic bias in terms of learning effects (Atkinson and Nevill, 1998), familiarization sessions were considered in only 38% of the studies with the most of them (60% of the studies) neglected this aspect and 2% provided limited details. This may increase sport-specific measurement bias and affect, thereafter, the accuracy of the test. The most adopted test-retest interval in 61% of the reviewed studies was 1 week. It should be noted that the test-retest interval should not be too short to avoid insufficient recovery between tests (Atkinson and Nevill, 1998) or too long to avoid being affected by participant's skill enhancement between the test and retest (Robertson et al., 2014). However, the exact test-retest interval is mainly dependent on the sport-specific test's characteristics in terms of complexity, duration, and type of effort required. Regarding the stability of testing conditions, most of the studies did not provide any (59%) or provided partial (13%) details. Again, this may affect the quality and accuracy of the sport-specific outcomes as different environmental conditions, for instance, may considerably influence testing results (Hachana et al., 2012).

### Reliability

Reliability is the ability of the testing protocol to provide similar outcomes from day to day when no intervention is used (Atkinson and Nevill, 1998). It is an important testing aspect as it provides indications about the biological as well as technical variation of the protocol (Bagger et al., 2003). From the three main aspects of reliability (i.e., test-retest, intra-, and inter-rater reliability, for in-depth details see Table 1), test-retest reliability represents the most studied sport-specific property (85% of studies that examined reliability) compared with intra/inter-rater reliability (15% of the studies) (Table 4). To effectively establish reliability, previous studies recommended determining both types of it i.e., relative and absolute reliability (Atkinson and Nevill, 1998; Weir, 2005; Impellizzeri and Marcora, 2009). To do so, a mixed statistical approach could be used, for instance, ICC which is indicative of the relative reliability of a test and SEM which is indicative of its absolute aspect (Atkinson and Nevill, 1998; Weir, 2005). Results of the current review showed that only few studies applied a mixed approach to examine both relative and absolute reliability of their sport-specific tests (40% of the studies that examined reliability). As this may constitute a limitation, upcoming investigations need to establish both types of reliability. On the other hand, a number of studies used other statistical approaches such as paired sample *t*-test (10% of the studies that addressed reliability). However, such an approach has been criticized in a previous review (Atkinson and Nevill, 1998) in the way that it does not provide any indication of random variation between tests. Additionally, Bland and Altman (1995) recommended paying attention to the interpretation of paired *t*-test results of reliability mainly because the detection of a significant difference is actually dependent on the amount of random variation between tests. Overall, to accurately establish sport-specific test's reliability, it is recommended to calculate both relative and absolute reliability by adopting appropriate statistical approaches. In addition, the other reliability aspects (i.e., inter/intra-rater) need to be investigated in conjunction with test-retest reliability. In that manner, a clear and accurate overview about the sport-specific test's reliability can be drawn. In fact, ensuring of test's reliability at first will enable the researcher to move on to check aspects related to validity and sensitivity. Otherwise, the testing protocol will be judged as non-valid. In this regard, Atkinson and Nevill (1998) argued that a measurement tool will never be valid if it provides inconsistent outcomes from repeated measurements.

### Validity and sensitivity

Content validity was established in all the reviewed studies. This is obvious since one of the current study's inclusion criteria is to deal with a sport-specific testing. Content validity was generally assumed (i.e., in 98% of the studies) by mainly referring to the specific literature and appraisal of the actual competition/combat requirements. However, only one study (Tabben et al., 2014) established this test's quality through a mixing of previous consultation with combat sports practitioners, coaches, sports scientists, and a review of the literature and competition requirements. Criterion validity was addressed in approximately half of the reviewed studies. The major part of these studies (95%) considered concurrent validity. Concurrent validity was generally studied by associating the sport-specific testing's outcome with a gold standard protocol (e.g., treadmill running test, cycle ergometer test). These gold standard tests are based on actions and thereafter involve muscle groups that are not combat sport specific, which may affect findings related to test's property (e.g., results reflective of a poor concurrent validity when the test reflect the true sport-related effort or findings indicative of good concurrent validity when the test did not reflect the true sport-related effort). From the whole eligible studies considered in this review, only one study addressed predictive validity (Lidor et al., 2005). This is particularly surprising in view of the critical importance of such a testing's property for coaches, strength and conditioning professionals, and combat sports athletes (Currell and Jeukendrup, 2008; Robertson et al., 2014). Therefore, future studies are encouraged to establish this important sport-specific testing's aspect.

Compared with criterion validity, construct validity received less attention in the literature (31% of the studies). Of note, only the discriminative side of construct validity was addressed by mainly comparing combat sports practitioners with a different competitive level and/or background (e.g., international vs. national level, elite vs. sub-elite) (Franchini et al., 1998, 2005, 2011b; Smith et al., 2000; Sterkowicz and Franchini, 2001; Tsolakis and Vagenas, 2010; Chaabène et al., 2012c; Chaabene et al., 2017b; Weichenberger et al., 2012; Del Vecchio et al., 2014; Chen et al., 2015; Tavra et al., 2016). To do so, the main statistical approach used were ROC analysis (Chaabène et al., 2012c; Chaabene et al., 2017b), independent sample *t*-test (Tsolakis and Vagenas, 2010; Weichenberger et al., 2012; Del Vecchio et al., 2014; Chen et al., 2015; Tavra et al., 2016), two-way repeated measures ANOVA (Franchini et al., 1998, 2011b; Smith et al., 2000; Sterkowicz and Franchini, 2001), and ANCOVA (Franchini et al., 2005). Nevertheless, as ROC analysis seems to be the more appropriate statistical approach to study discriminative ability of a test (Chaabène et al., 2012c; Chaabene et al., 2017b; Castagna et al., 2014), future investigations are recommended to use this approach. Regarding convergent validity, it was not studied in any of the reviewed studies. This seems to be due to the fact that creating a new sport-specific test is mainly due to a gap in the literature so there is no previous protocol to compare with the new one (Streiner and Norman, 2005). It is noteworthy that 8% of the studies (Weichenberger et al., 2012; Del Vecchio et al., 2014; Chaabene et al., 2017b) addressed, at least, one aspect of the three sport-specific properties (i.e., content, criterion, and construct validity). This observation may constitute another gap in the literature because, to be considered valid and applicable, a sport-specific test should cover the whole validity aspects (i.e., content, criterion, and construct validity). Thereafter, a particular focus in the future investigations should be given to examining all validity properties of sport-specific performance testing in Olympic combat sports.

Another important property related to sport-specific testing is the sensitivity (Currell and Jeukendrup, 2008; Impellizzeri and Marcora, 2009). Findings of the current review showed that only 4 studies examined this aspect (Chaabène et al., 2012c; Chaabene et al., 2017b; Tabben et al., 2014; da Silva Santos and Franchini, 2016). Additionally, despite its importance from a practical point of view, the minimal detectable change was investigated in only 3 studies (Chaabène et al., 2012a; Chaabene et al., 2017b; Tabben et al., 2014). Therefore, more research dealing with these two determinant aspects are required.

### Utility and limitations

In reviewing studies that aimed to validate sport-specific tests, thorough details about the applicability (i.e., whether it is easy to administer and scored) and the limits of the test in question were expected. However, most of the selected studies (64%) either partially detailed or ignored this valuable aspect. Details related to sport-specific test background were either partially or even ignored in 28% of the studies (Franchini et al., 1998, 2005; Smith et al., 2000; Tsolakis and Vagenas, 2010; Obminski et al., 2011; Santos et al., 2011; Sertić et al., 2011; Shiyan, 2011; Oliveira et al., 2015; Sogabe et al., 2015; Tavra et al., 2016). Therefore, future investigations should pay attention to these central sport-specific tests' aspects.

## Limitations

Because of the variety of statistical approaches used to assess sport-specific measurements properties, it was not possible to perform any meta-analysis (Robertson et al., 2014). Additionally, compared with other sporting activities such as team sports, scientific contributions on combat sports in indexed journals are limited, with studies mainly published in non-indexed journals (i.e., gray literature) or remain even unpublished. Therefore, the stringent search approach adopted in this review has neglected information available to coaches in specific technical magazines and websites.

## Conclusions and future recommendations

Establishing valid sport-specific tests that assess the actual physical fitness and/or physiological attributes of Olympic combat sports practitioners still one of the major concerns for sports sciences scholars. After reviewing 39 studies in different Olympic combat sports disciplines (e.g., karate, taekwondo, amateur boxing, judo, wrestling, and fencing), several methodological gaps have been pointed-out. These limits may prevent sport-specific testing from being widely used. These limitations are mainly related to the small sample size, backgrounds of participants, being elite level in most of the studies, sex (mainly males), lack of details about the inclusion/exclusion criteria in most of the studies, lack of familiarization session prior to testing, and paucity of details about stability of testing conditions. Additionally, both types of reliability (e.g., relative and absolute) have rarely been addressed in the reviewed studies and the available results showed reliability levels ranging from poor to excellent. Moreover, despite its critical importance, predictive validity was reported in only one study. Similarly, compared with criterion validity, construct validity received less attention by researchers. Studies addressing, at least, one aspect of the three main validity properties are limited. All these concerns may limit the applicability, generality, and accuracy of outcomes of sport-specific testing in Olympic combat sports. Additional research should adopt more strict validation procedures by addressing reliability, validity, and sensitivity in the application and description of sport-specific performance tests in Olympic combat sports. Additionally, predictive validity should receive more attention in future research.

## Author contributions

HC: Worked on study design, data collection, data analysis, and manuscript preparation. YN: Worked on data collection, data analysis, and manuscript preparation. RB: Assisted on data collection and analysis and worked on manuscript preparation. LC: Assisted in study design and worked on manuscript preparation. EF: Worked on manuscript preparation. OP: Worked on manuscript preparation. HH: Worked on manuscript preparation. UG: Data analysis and manuscript preparation.

### Conflict of interest statement

The authors declare that the research was conducted in the absence of any commercial or financial relationships that could be construed as a potential conflict of interest.
